# RETRACTED: Godugu et al. Anti-Cancer Activities of Thyrointegrin α_v_β_3_ Antagonist Mono- and Bis-Triazole Tetraiodothyroacetic Acid Conjugated via Polyethylene Glycols in Glioblastoma. *Cancers* 2021, *13*, 2780

**DOI:** 10.3390/cancers16101880

**Published:** 2024-05-15

**Authors:** Kavitha Godugu, Mehdi Rajabi, Shaker A. Mousa

**Affiliations:** The Pharmaceutical Research Institute, Albany College of Pharmacy and Health Sciences, Rensselaer, NY 12208, USAm.rajabi.s@gmail.com (M.R.)

The journal retracts the article, “Anti-Cancer Activities of Thyrointegrin α_v_β_3_ Antagonist Mono- and Bis-Triazole Tetraiodothyroacetic Acid Conjugated via Polyethylene Glycols in Glioblastoma” [1].

During a recent investigation, the Albany College of Pharmacy and Health Sciences determined that multiple images were falsified or fabricated in this paper [1]. The misconduct findings are associated with the manipulation, re-use, and relabeling of images, and since the authors are members of Albany College, the college has requested the retraction of the article.

A correction [1] was submitted. However, the authors did not sufficiently explain the issues with the original images to the investigation committee, creating cause for concern regarding the validity of the experiments.

This retraction was approved by the Editor-in-Chief of the journal *Cancers*. 

The authors did not agree with this retraction.
